# Neighborhood-level sociodemographics and kindergarten children’s developmental vulnerability, pre- and post-COVID-19 in Canada

**DOI:** 10.23889/ijpds.v9i5.2708

**Published:** 2024-09-18

**Authors:** Caroline Reid-Westoby, Ashley Gaskin, Amanda Offord, Eric Duku, Barry Forer, Marc Jambon, Magdalena Janus

**Affiliations:** 1 McMaster University; 2 University of British Columbia; 3 Wilfrid Laurier University

A child’s environment and early life experiences play an important role in shaping their development. The COVID-19 pandemic altered many aspects of everyday life for young children in Canada, and it has been argued that these changes did not impact all children equally. The current study explored the association between early child development and neighborhood sociodemographic indices (e.g., income, deprivation) before and after the onset of the COVID-19 pandemic. The Early Development Instrument (EDI) data, measuring child development and school readiness in kindergarten, were collected in 7 Canadian provinces and 1 territory from 2017-2020 (pre-COVID) and 2020-2023 (post-COVID) and were linked with neighborhood-level socioeconomic indices from 2016. We compared the gradients in development in a pre- (n=293,700) and a post-COVID (n=246,305) cohort of Canadian children. Rates of developmental vulnerability, as measured by the EDI, were examined for the sociodemographic variables in both the pre- and post-COVID cohorts. The overall vulnerability increased nationally from 27.3% in the pre- to 28.0% in the post-COVID cohort. A gradient in vulnerability rates for all the socioeconomic indices was found, with children in the highest quintiles, representing greater deprivation, having greater odds of being vulnerable than their peers in the lowest, least deprived quintile. Contrary to expectation, the magnitude of the gradient was very similar in both cohorts, with some jurisdictional differences. The largest gradient was found for average neighborhood income. These findings add the context of social determinants of health to the understanding of the impact of the COVID-19 pandemic on young children.

